# “Community Over Commercialization”: Help Us Keep GHSP Open

**DOI:** 10.9745/GHSP-D-24-00608

**Published:** 2025-08-14

**Authors:** Sonia Abraham, Natalie Culbertson, Stephen Hodgins, Ruwaida M. Salem

**Affiliations:** a Formerly with Johns Hopkins Center for Communication Programs, Baltimore, MD, USA.; b School of Public Health, University of Alberta, Edmonton, Canada. Editor-in-Chief of Global Health: Science and Practice Journal, Baltimore, MD, USA.; c Johns Hopkins Center for Communication Programs, Baltimore, MD, USA.

Since its inception in 2013, *Global Health: Science and Practice* (GHSP) has aimed to bridge the gap between knowledge and action in global health programs by embracing diverse forms of knowledge and learning to better understand what works and what doesn’t in real-world settings. We also have provided an equitable publishing platform to ensure that knowledge is cultivated and disseminated as a global good, not a commodity.^1^^,^^2^ With financial support from the U.S. Agency for International Development (USAID) and additional, limited support from organizations, such as the Gates Foundation, the Doris Duke Charitable Foundation, and People that Deliver, for publishing supplements, GHSP has remained one of the few global health journals that is truly open access. The journal has not charged any article processing fees to authors, regardless of location, affiliation, or article type, and has not imposed subscription fees on readers.

By prioritizing “community over commercialization,”^3^^,^^4^ GHSP has fostered a global community of health practitioners, program managers, decision-makers, and policymakers globally who are better connected to each other and to evidence on what works and under what conditions. GHSP’s model has helped to strengthen programs and amplify their impact to facilitate lasting change. Now, this model—and even the journal itself—is at risk. In February 2025, amid the broad terminations of USAID-funded global health projects, USAID ended its support for GHSP.

As the publisher of GHSP, the Johns Hopkins Center for Communication Programs (CCP), along with the editor-in-chief and associate editors, remains committed to maintaining GHSP as an independent journal. CCP is actively exploring ways to achieve this without imposing fees on authors or readers. We know that the journal fulfills an essential role for public health practitioners and researchers worldwide—a role that has become even more pronounced in today’s global context. GHSP provides a critical space for documenting and sharing the experiences of governments, nongovernmental organizations, the private sector, international organizations, funding agencies, and communities as they navigate unprecedented change to identify what works and what doesn’t. Our readers, authors, and reviewers share this commitment, with many asking what they can to do to ensure GHSP’s future during these times of transition.

In this editorial, we share insights into how we operate as a journal, highlight data on our impact, and outline plans to sustain GHSP, including how you can help.

## HOW WE OPERATE

Four years ago, as calls for decolonizing global health and publishing grew louder, GHSP recognized the critical role of journals as unintentional knowledge gatekeepers.^5^ We committed to reexamining how we operate and finding better ways to fulfill our mission of promoting knowledge equity. To address epistemic injustice^6^ that is often present in collaborations between low- and middle-income countries (LMICs) and high-income countries, we set out to amplify the voices and perspectives of researchers and practitioners in LMICs who make valuable contributions to global health programming, research, and publishing.^7^^–^^9^

We began with an internal review of operations to address power asymmetries^10^^,^^11^ and strengthened our editorial team to better reflect gender, socioeconomic, and geographic diversity.^12^^,^^13^ We also changed our editorial policies and procedures to foster inclusivity and equitable partnerships in global health research and publishing.^14^^,^^15^

Recognizing that publishing exclusively in English creates barriers to knowledge access, we began offering the entire GHSP website and all articles in more than 100 languages through the Google Translate widget.^16^^,^^17^ While we acknowledge the limitations of machine translation, we also publish professional translations of abstracts and full articles into French, Spanish, or Portuguese, when possible.

We also are mindful of the under-representation of health practitioners and policymakers from LMICs in the peer-reviewed literature—particularly those outside academic systems where publication frequency is tied to career advancement.^18^^–^^20^ To address this, we have provided pre-peer review feedback and writing support to potential authors whose articles offer valuable perspectives on global health programming but who may lack the time, skills, resources, or experience to publish peer-reviewed journal articles.

Finally, even before USAID’s terminations, we had started exploring partnerships with LMIC institutions to strengthen to the journal’s strategic direction and governance, expand our reach to LMIC-based authors and readers, and provide more robust capacity-strengthening support to authors.

## OUR IMPACT

Our efforts to foster a diverse global health community have been successful. GHSP authors hail from 143 countries, 99 of which are LMICs. Our reviewers are from 101 countries, of which 78 are LMICs. Readers from more than 210 countries—56% from Africa and Asia—have accessed our articles more than 1.5 million times in the past year. Between January 2019 and September 2024, the proportion of authors from LMICs who published in GHSP (regular and supplement issues) increased from 40% to 63%, first authors from LMICs increased from 17% to 47%, and articles with at least one LMIC author increased from 61% to 90%.

Perhaps an even stronger indicator of GHSP’s impact, beyond conventional academic metrics, is how our readers worldwide have engaged with our content. To date, we have published more than 1,000 articles, which have been referenced more than 13,100 times in 454 policy documents, briefs, news articles, blogs, and social media channels (see Box). These citations illustrate how our readers use, share, and apply GHSP content to inform health policies, programs, and practices that drive local and global health goals.

BOX GHSP by the Numbers

- 1,000+ published articles, with ∼50% receiving 10+ citations
- 1.53 million article accesses per year
- 13,100+ references in policy documents, news articles, and social media
- 80% of readers said GHSP improved their understanding of global health
- 78% of readers said they used GHSP to enhance programs, research, and policy

To delve deeper into how our readers use our content, we surveyed authors, reviewers, and advisory board members in April 2024, receiving more than 800 responses from 28 countries, with 55% from LMICs. Ninety-six percent of the respondents confirmed that GHSP is relevant to their work, citing its **unique emphasis on practical program knowledge over theoretical research**, compared with other global health journals.

*What really attracted me [to GHSP] is the focus on implementation. Usually, global health journals cover the problem and prospective solutions but not an actual strategic plan and the implementation of that plan.* … *It*’*s not just about something that can be done, but something that has been done.* —GHSP author, Pakistan

*The journal helps to articulate public health practices in resource-limited settings by documenting best practices, lessons learned, and challenges in addressing public health gaps.* —GHSP author, Sudan

*GHSP has carved out its niche by emphasizing the translation of evidence into practice. GHSP prioritizes research that is not only rigorous but also directly applicable to real-world health interventions and programs.* —GHSP author, Bangladesh

Approximately 80% of respondents reported that GHSP improved their understanding of global health programs, and **78% indicated that they had applied information from GHSP articles to their work**. Of those, 571 respondents shared that they had used GHSP content to guide research, design or improve projects or programs, revise training materials, or update or create new public health policies and guidelines. More details from the survey are summarized in the accompanying graphic.

Some respondents reported specific examples of how they used GHSP content.

*I have used what I have learned from [GHSP] to directly improve the health programming that I implement [and] share information with national-level technical working groups and colleagues.* —GHSP reader, Uganda

*We used the guide and instructions from GHSP to implement a new guideline, which empowers the nurses to help with early diagnosis of malnutrition.* —GHSP reader, Mozambique

Respondents also shared the **importance of being able to read, publish, and share content in GHSP freely.**

*GHSP gives more premium to scientific knowledge than monetary gains from authors; hence, it does not charge authors for publication. It is obvious that some relevant works have not come to the public knowledge due to the high cost of publication charged by other journals. But to GHSP, it is different.* —GHSP author and reader, Nigeria

*It is the only journal with status that is truly flattening the power dynamics of global health engagement such that everyone has a fair chance and is able to create new things from shared ideas.* —GHSP reader, United Kingdom

*Since many [global health] researchers focusing on implementation have limited publication budgets, the fee structure also fills a critical role in getting implementation research to press and readable to global audiences.* —GHSP author, Ethiopia

## LOOKING AHEAD

GHSP has been a trusted resource for 12 years, benefiting program implementers, researchers, and policymakers, but it now faces a critical juncture. The termination of USAID funding has placed the journal at risk, forcing us to temporarily halt operations, including publishing accepted articles and considering new submissions.

With this editorial and the accompanying articles in our August 2025 issue, CCP reaffirms its commitment to sustaining GHSP. Over the next several months, we will process and publish previously accepted articles. At this time, we will delay new author manuscript submissions to ensure we have time to process the backlog of previously submitted manuscripts. CCP will also actively seek funding to secure the operating budget necessary to maintain GHSP’s open-access model for readers and authors.

Although CCP has been exploring different revenue-generation models and cost-saving measures for the journal for some time, even before the USAID termination, donor funding remains essential. Without it, we risk being forced to charge article processing fees to some or all authors—an outcome we are committed to avoiding if at all possible.

What sets GHSP apart from other journals, making it a strategic and worthwhile investment?

- Free and open access for *all* authors *and* readers
- Rigorous, high-quality peer review to deliver access to practical and actionable content
- Personalized and helpful editorial guidance and support for authors
- Direct investment in amplifying the voices of researchers, program implementers, and institutions in LMICs

Ultimately, donor support for GHSP is a **commitment to the global community**, ensuring the continued publication of high-quality articles that give thoughtful consideration to what works, and under what conditions, in health programs worldwide. Historically, our budget yielded a cost of only $0.32 per article access—a very modest investment for a publication reaching hundreds of thousands of frontline public health professionals with critical, practical, and timely information.

**If your foundation, corporation, or organization is interested in supporting GHSP, please reach out to CCP’s Business Development team at ccpbd@jhu.edu.** CCP welcomes funding commitments of all sizes and durations, from short-term contributions to long-term partnerships.

**We also have launched a fund for individual donations—small and large—to Keep GHSP Open**. Please visit https://secure.jhu.edu/form/JohnsHopkinsCCP, select “The Johns Hopkins Center for Communication Programs” from the Gift Designation drop-down menu. Until September 30, your donation will automatically be directed to help CCP keep GHSP open to readers and authors, ensuring that lifesaving, practical information on effective programs reaches decision-makers around the world.

You also can support GHSP in other ways:

- **Share this editorial** with your network, or anyone who values open access publishing and global health programs, using the hashtag **#KeepGHSPOpen**.
- **Tell your story** of how GHSP has made an impact on you and your work with the hashtag **#KeepGHSPOpen**.
- **Introduce us** to someone in your network who wants to make an impact.

Without GHSP, many health practitioners around the world would not have access to practical evidence they need to make their programs more effective, to adapt to evolving challenges, and to share their expertise with their peers. By working together, we can ensure GHSP remains a high-quality global good that is committed to community over commercialization.

## Notes

Peer Reviewed

## References

[1] Global Summit on Diamond Open Access. Conclusions and Way Forward. Accessed December 4, 2024. https://globaldiamantoa.org/wp-content/uploads/2023/10/202310-Global-Summit-Conclusions-Way-Forward.pdf

[2] Vervoort D, Ma X, Bookholane H. Equitable open access publishing: changing the financial power dynamics in academia. Glob Health Sci Pract. 2021;9(4):733–736. 10.9745/GHSP-D-21-00145. 34933971 PMC8691877

[3] Community over commercialization. Open Access Week 2024. International Open Access Week. Accessed December 4, 2024. https://www.openaccessweek.org/

[4] Fernández Pinto M. Open science for private interests? How the logic of open science contributes to the commercialization of research. Front Res Metr Anal. 2020;5:588331. 10.3389/frma.2020.588331. 33870052 PMC8025975

[5] Khan SA. Decolonising global health by decolonising academic publishing. BMJ Glob Health. 2022;7(3):e007811. 10.1136/bmjgh-2021-007811. 35301234 PMC8931802

[6] Bhakuni H, Abimbola S. Epistemic injustice in academic global health. Lancet Glob Health. 9(10):e1465–e1470. 10.1016/S2214-109X(21)00301-6. 34384536

[7] Boum Ii Y, Burns BF, Siedner M, Mburu Y, Bukusi E, Haberer JE. Advancing equitable global health research partnerships in Africa. BMJ Glob Health. 2018;3(4):e000868. 10.1136/bmjgh-2018-000868. 30167335 PMC6112391

[8] Parker M, Kingori P. Good and bad research collaborations: researchers’ views on science and ethics in global health research. PLoS One. 2016;11(10):e0163579. 10.1371/journal.pone.0163579. 27737006 PMC5063577

[9] Matenga TFL, Zulu JM, Corbin JH, Mweemba O. Contemporary issues in north–south health research partnerships: perspectives of health research stakeholders in Zambia. Health Res Policy Syst. 2019;17(1):7. 10.1186/s12961-018-0409-7. 30646902 PMC6334387

[10] Abraham S, Hodgins S, Saad A, Fabic MS. What is Global Health: Science and Practice doing to address power imbalances in publishing? Glob Health Sci Pract. 2020;8(3):325–326. 10.9745/GHSP-D-20-00453. 33008848 PMC7541102

[11] Nafade V, Sen P, Pai M. Global health journals need to address equity, diversity and inclusion. BMJ Glob Health. 2019;4(5):e002018. 10.1136/bmjgh-2019-002018. 31750004 PMC6830051

[12] Ricca J, Abraham S, Waiswa P, Culbertson N, Hodgins S. Toward decolonizing knowledge production in global public health: results of a multi-level intervention to improve equity of authors at a global health journal. Knowledge Management Dev J. 2023;17(1/2):42–56. Accessed December 4, 2024. https://www.km4djournal.org/index.php/km4dj/article/view/534/656

[13] Manan MR, Nawaz I, Rahman S, et al. Diversity, equity, and inclusion on editorial boards of global health journals. Asian Bioeth Rev. 2023;15(3):209–239. 10.1007/s41649-023-00243-8. 37399000 PMC10018626

[14] Reflexivity policy and checklist. Global Health Science and Practice. Accessed December 4, 2024. https://www.ghspjournal.org/content/instructions-authors#Reflexivity

[15] Taylor M, Heinz E, Gondwe M, et al. Authorship reflexivity statements: additional considerations. BMJ Glob Health. 2024;9(1): e014743. 10.1136/bmjgh-2023-014743. 38176744 PMC10773370

[16] Hommes F, Monzó HB, Ferrand RA, et al. The words we choose matter: recognising the importance of language in decolonising global health. Lancet Glob Health. 2021;9(7):e897–e898. 10.1016/S2214-109X(21)00197-2. 34143986

[17] Adebisi YA, Jimoh ND, Ogunkola IO, Ilesanmi EA, Elhadi YAM, Lucero-Prisno DE III. Addressing language inequities in global health science scholarly publishing. J Med Surg Public Health. 2024;2:100038. 10.1016/j.glmedi.2023.100038

[18] Oronje RN, Mukiira C, Kahurani E, Murunga V. Training and mentorship as a tool for building African researchers’ capacity in knowledge translation. PLoS One. 2022;17(3):e0266106. 10.1371/journal.pone.0266106. 35358255 PMC8970368

[19] Kalbarczyk A, Rodriguez DC, Mahendradhata Y, et al. Barriers and facilitators to knowledge translation activities within academic institutions in low- and middle-income countries. Health Policy Plan. 2021;36(5):728–739. 10.1093/heapol/czaa188. 33661285 PMC8173595

[20] Busse CE, Anderson EW, Endale T, et al. Strengthening research capacity: a systematic review of manuscript writing and publishing interventions for researchers in low-income and middle-income countries. BMJ Glob Health. 2022;7(2):e008059. 10.1136/bmjgh-2021-008059. 35165096 PMC8845213

